# Enhanced Dichotic Listening and Temporal Sequencing Ability in Early-Blind Individuals

**DOI:** 10.3389/fpsyg.2022.840541

**Published:** 2022-05-10

**Authors:** Eun Bit Bae, Hyunsook Jang, Hyun Joon Shim

**Affiliations:** ^1^Department of Otorhinolaryngology-Head and Neck Surgery, Nowon Eulji Medical Center, Eulji University, Seoul, South Korea; ^2^Division of Speech Pathology and Audiology, Research Institute of Audiology and Speech Pathology, Hallym University, Chuncheon, South Korea

**Keywords:** early visual deprivation, dichotic listening, temporal sequencing ability, speech in noise, central auditory processing, visual cortex

## Abstract

Several studies have reported the better auditory performance of early-blind subjects over sighted subjects. However, few studies have compared the auditory functions of both hemispheres or evaluated interhemispheric transfer and binaural integration in blind individuals. Therefore, we evaluated whether there are differences in dichotic listening, auditory temporal sequencing ability, or speech perception in noise (all of which have been used to diagnose central auditory processing disorder) between early-blind subjects and sighted subjects. The study included 23 early-blind subjects and 22 age-matched sighted subjects. In the dichotic listening test (three-digit pair), the early-blind subjects achieved higher scores than the sighted subjects in the left ear (*p* = 0.003, Bonferroni’s corrected α = 0.05/6 = 0.008), but not in the right ear, indicating a right ear advantage in sighted subjects (*p* < 0.001) but not in early-blind subjects. In the frequency patterning test (five tones), the early-blind subjects performed better (both ears in the humming response, but the left ear only in the labeling response) than the sighted subjects (*p* < 0.008, Bonferroni’s corrected α = 0.05/6 = 0.008). Monosyllable perception in noise tended to be better in early-blind subjects than in sighted subjects at a signal-to-noise ratio of –8 (*p* = 0.054), the results at signal-to-noise ratios of –4, 0, +4, and +8 did not differ. Acoustic change complex responses to/ba/in babble noise, recorded with electroencephalography, showed a greater N1 peak amplitude at only FC5 electrode under a signal-to-noise ratio of –8 and –4 dB in the early-blind subjects than in the sighted subjects (*p* = 0.004 and *p* = 0.003, respectively, Bonferroni’s corrected α = 0.05/5 = 0.01). The results of this study revealed early-blind subjects exhibited some advantages in dichotic listening, and temporal sequencing ability compared to those shown in sighted subjects. These advantages may be attributable to the enhanced activity of the central auditory nervous system, especially the right hemisphere function, and the transfer of auditory information between the two hemispheres.

## Introduction

Because congenitally blind or early-blind individuals depend exclusively upon auditory sensory cues, without visual cues when communicating, their auditory processing can develop differently from that of sighted subjects. Several studies have reported the better performance of early-blind subjects over sighted subjects in speech memory (Amedi et al., 2003), pitch discrimination in pure tone (Gougoux et al., 2004; Shim et al., 2019), temporal resolution (Stevens and Weaver, 2005; Shim et al., 2019), ultrafast speech comprehension (Hertrich et al., 2013), and dichotic listening (Hugdahl et al., 2004). Some studies found impaired sound localization abilities in early-blind subjects, especially in the sound localization task in the vertical plane (Zwiers et al., 2001; Lewald, 2002) or the performance of more complex tasks requiring a metric representation of the auditory space (Gori et al., 2010, 2014; Finocchietti et al., 2015; Vercillo et al., 2016). However, a recent study showed enhanced spatial hearing abilities in early-blind subjects and claimed that vision is not a prerequisite for developing an auditory sense of space (Battal et al., 2020). The difference in auditory performance in early-blind subjects compared to sighted subjects is presumed to be due to plastic changes in the central auditory system. Abundant neuroimaging evidence supports the theory that the cerebral cortex experiences compensatory plasticity after visual deprivation. The visual cortices of blind subjects have been shown to be recruited following auditory signals (Weeks et al., 2000; Voss et al., 2008; Gougoux et al., 2009). However, few studies have compared the auditory functions of both hemispheres or evaluated interhemispheric transfer and binaural integration in blind individuals (Hugdahl et al., 2004). Several behavioral tests have been developed to evaluate the central auditory function in each targeted process, and they have been used to diagnose central auditory processing disorder. In most listening environments, both ears do not receive the same signal at the same time, so the brain must be able to integrate the potentially competing information from both ears. A dichotic listening test assesses the auditory function in the left and right hemispheres separately and evaluates the biaural integration that occurs through information transfer from the right hemisphere to the left hemisphere (Musiek, 1983; Jutras et al., 2012; Rajabpur et al., 2014; Fischer et al., 2017). The frequency pattern test measures frequency discrimination and temporal sequencing ability for sound and is known to be highly sensitive to and specific for lesions in the corpus collosum, which is responsible for connecting the information between both hemispheres (Musiek, 1994; Marshall and Jones, 2017). Here, we used the dichotic listening and frequency pattern tests to compare early-blind subjects and sighted subjects.

Moreover, the ability to process low-redundancy speech, such as speech perception in noise, can be one way to explore the central auditory system. It has not been concluded whether there are differences in speech perception in noise between early-blind individuals and sighted individuals. Several studies that compared speech perception between early-blind and sighted subjects have shown varying results, depending on the experimental conditions (Gougoux et al., 2009; Menard et al., 2009; Hertrich et al., 2013; Arnaud et al., 2018). In a previous study, we confirmed that early-blind subjects showed greater frequency and temporal resolution insofar as they scored better on spectral discrimination and modulated detection thresholds than sighted subjects (Shim et al., 2019). Auditory spectral resolution and temporal resolution are the fundamental aspects of speech perception. However, there was no difference of two-syllable speech perception in noise between the blind subjects and sighted subjects. When interpreting the results for the auditory performance of blind subjects in our previous study, together with other results in the literature, we wondered why there was no consistent result for speech perception in noise, even though frequency and temporal resolution were consistently better in blind subjects in previous studies. We speculated that the dichotic listening and frequency pattern tests may provide clues to resolving this question. We hypothesized that plastic changes in the central auditory system after visual deprivation affect the two cerebral hemispheres differently, and affect the interhemispheric transfer from the right to the left cortex in early-blind individuals.

In our previous study (Shim et al., 2019), two-syllable words were presented in background noise, and we assume that this condition involves the listener’s ability to achieve auditory closure with redundancy cues, in addition to his/her ability to discriminate speech cues (Aylett and Turk, 2004). To minimize the redundancy cues in speech perception and to force the listener to focus on speech cues itself, we used monosyllabic words consisting of consonant–vowel (CV) or consonant–vowel–consonant (CVC) sequences in the present study.

In addition to the monosyllabic speech perception test, we recorded the acoustic change complex (ACC) in response to/ba/in babble noise. The ACC is an auditory evoked potential (P1-N1-P2) that can be elicited by the listener’s ability to detect a change in an ongoing sound in passive listening conditions (Martin and Boothroyd, 1999; Tremblay et al., 2003).

The purpose of this study was to evaluate whether there are differences in dichotic listening, auditory temporal sequencing ability, and speech perception in noise between early-blind subjects and sighted subjects. In the current study, the early-blind subjects were limited to those who were blind at birth or who became blind within 1 year of birth because late-blind subjects showed lower levels of plasticity in spectral resolution than early-blind subjects in our previous study (Shim et al., 2019).

## Materials and Methods

### Subjects

The study population included a group of 23 early-blinded subjects aged 19–40 years (29.57 ± 5.03 years, male:female [M:F] = 12:11) and an age-matched control group of 22 sighted subjects (28.59 ± 4.66 years, M:F = 11:11). There were no significant differences between the two groups in age (*p* > 0.05). All the subjects were right-handed, aged < 40 years, with normal symmetric hearing thresholds (≤20 dB hearing level at 0.25, 0.5, 1, 2, 3, 4, and 8 kHz), and the pure tone averages did not differ significantly between the two groups (*p* > 0.05). They had no other neurological or otological problems. In the blind group, only those who were blind at birth or who became blind within 1 year of birth, who were classified in categories 4 and 5 according to the 2006 World Health Organization guidelines for the clinical diagnosis of visual impairment (category 4, “light perception” but no perception of “hand motion”; category 5, “no light perception”), were included. Table 1 shows the characteristics of the blind subjects. We confirmed the normal cognitive abilities of all the subjects using the Korean Mini-Mental State Examination. The Mini-Mental State Examination was used except for vision-related items, and no difference was detected between the two groups (*p* > 0.05). The study was conducted in accordance with the Declaration of Helsinki and the recommendations of the Institutional Review Board of Nowon Eulji Medical Center, with written informed consent from all subjects. Informed consent was obtained verbally from the blind subjects in the presence of a guardian or third party. The subjects then signed the consent form, and a copy was given to them.

**TABLE 1:** Clinical characteristics for the early-blind (EB) subjects.

Subject	Age (yeas)	Onset	Sex	Visual acuity	Cause of blindness
EB-1	37	Birth	F	No light perception	Optic nerve atrophy
EB-2	29	Birth	M	No light perception	Persistent hyperplastic primary vitreous
EB-3	25	Birth	M	No light perception	Retinopathy of prematurity
EB-4	33	Birth	F	Light perception	Microphthalmos
EB-5	31	Birth	F	Light perception	Retinoblastoma
EB-6	20	Birth	F	Light perception	Corneal opacity
EB-7	22	Birth	M	No light perception	Optic nerve atrophy
EB-8	33	Birth	M	Light perception	Optic nerve atrophy
EB-9	31	Birth	M	Light perception	Retinopathy of prematurity
EB-10	30	Birth	F	Light perception	Congenital glaucoma
EB-11	37	Birth	M	Light perception	Optic nerve atrophy
EB-12	22	Birth	M	No light perception	Retinal detachment
EB-13	25	Birth	M	Light perception	Microphthalmos
EB-14	29	Birth	F	Light perception	Retinopathy of prematurity
EB-15	34	Birth	F	No light perception	Optic nerve atrophy
EB-16	27	Birth	M	Light perception	Unknown
EB-17	27	Birth	F	No light perception	Retinopathy of prematurity
EB-18	29	Birth	M	Light perception	Unknown
EB-19	25	Birth	F	Light perception	Optic nerve atrophy
EB-20	36	Birth	M	Light perception	Optic nerve atrophy
EB-21	31	Birth	M	No light perception	Optic nerve atrophy
EB-22	29	Birth	F	No light perception	Optic nerve atrophy
EB-23	38	Birth	F	No light perception	Congenital cataract

*M, Male; F, Female.*

### Behavioral Tests

Three behavioral tests were used to evaluate central auditory processing: the dichotic digit test, the frequency pattern test, and the monosyllable perception in noise test. The digit span test was also conducted to examine the effect of working memory on central auditory processing. All tests were conducted in a sound-proofed room with an audiometer (Madsen Astera 2; GN Otometrics, Taastrup, Denmark) and inserted earphones (ER-3A; Etymotic Research, Inc., Elk Grove Village, IL, United States). Behavior tests were presented in the same order: digit span test, dichotic digit test, frequency pattern test—humming, frequency pattern test—labeling, and monosyllable perception test. Each frequency pattern test was performed first on the right ear and then on the left ear. The monosyllable perception test was conducted randomly at five signal-to-noise ratios (SNRs).

#### Dichotic Digit Test

The dichotic digit test, developed in Korean by Jeon and Jang (2009), was used to evaluate dichotic listening ability. The Korean dichotic digit test, consisting of one-, two-, and three- digit pairs, was standardized (Jang et al., 2014). In this study, two subtests were performed, the dichotic two-digit test and the dichotic three-digit test, which previous studies have suggested are appropriate for clinical use in young adults with normal hearing (Jeon and Jang, 2009; Jang et al., 2014). Each subtest consisted of 20 items with a 500 ms interdigit interval and a 5 s interstimulus interval. The digit stimuli were presented to the bilateral ears simultaneously at 60 dB HL, and the subjects responded verbally in the free-recall mode, in which they were asked to say all the digits they heard in both ears. The numbers of correct responses in the right and left ears were calculated individually in both subtests.

#### Frequency Pattern Test

The frequency pattern test measures temporal sequencing ability and assesses the integrity of the hemispheric and interhemispheric transfer of neural information (Musiek, 1994). The frequency pattern test was presented in three sequential tones, which were “high” (1,122 Hz) or “low” (880 Hz) in frequency. The test was performed in each ear and the subjects were instructed to respond by humming or labeling. The subjects were instructed to hum or label (e.g., high–low–high–high–low) in response to the given stimulus, which was presented monaurally at 60 dB HL. The humming condition, which imitates the pitch pattern of the tone heard, evaluates the function of the right hemisphere, which is mainly responsible for the recognition of acoustic contour and patterns. In contrast, the labeling condition, which is a verbal response to the pitch patterns, reflects the integrity of the left hemisphere, which dominates speech recognition (Kimura, 1961; Blumstein and Cooper, 1974). In this study, the test complexity was modified from three to five sequential tones, with reference to the study of Yoon et al. (2019), which reported the differences in the frequency pattern test performance of young adults with normal hearing. Each tone was 150 ms in duration, with a 10 ms rise–fall time and a 200 ms intertone interval. The frequency pattern test consisted of 60 items, which were divided into 30 items each for the humming and labeling responses. The order of the high–low tone sequence was controlled in such a way that no more than three identical tones were presented in a row. A total of 30 possible patterns were presented equally to the four conditions (left-humming, left-labeling, right-humming, and right-labeling) of 15 items. The numbers of correct responses (15 scores for each condition) in the right and left ears were calculated individually for both the humming and labeling responses.

#### Monosyllable Perception in Noise Test

The monosyllable perception in noise test was performed at five SNRs (+8, +4, 0, –4, and –8) using five lists, each containing 25 Korean monosyllabic words, which were spoken by a male speaker, and eight-talker babble noise. The mixture of the target word and the noise stimuli was presented monaurally to the test ear, and the subjects were asked to repeat the words while ignoring the noise. The noise level was fixed at 70dB SPL and the level of the target words was varied. The number of correct responses for a total of 25 words under each of the SNR conditions was measured. For the sighted subjects, the test was performed under both auditory-only (AO) and audio–visual (AV) conditions. For the AV condition, video clips that included the speaker’s face and sound were presented through a monitor and a loudspeaker located 1 m in front in a sound-attenuating booth, and for the AO condition, only the sound signal was presented. The main comparison was between the performance of early-blind subjects and that of sighted subjects under the AO condition, so the monosyllable perception in noise was first measured under the AO condition and then under the AV condition in the sighted subjects.

#### Digit Span Test

All digit span tests consisted of digits from 1 to 9, and the digit sets presented increased consecutively from three to 10 digits. Digit sets of the same numbers were presented twice. The threshold of the digit span test was determined to be at least two incorrect responses to the previous digit series. The sets of digits were presented to the bilateral ears simultaneously at 60 dB HL, with a 1 s interval between sets, and the subjects were asked to repeat the set of digits in a forward manner. The highest total score for the test was 16, and the maximum number of digits was 10 (Supplementary Material 1).

### Electrophysiological Methods

#### Acoustic Change Complex Stimuli

The first 400 ms of the stimulus consisted of babble noise and the last 400 ms contained babble noise plus/ba/sound in five SNR conditions (–8, –4, 0, +4, and +8 dB) followed by an inter-stimulus interval of 150 ms. Each SNR condition involved 100 random presentations of babble noise –/ba/with the same noise stimuli. The stimuli were presented to the bilateral ears simultaneously at 60 dB HL.

#### Procedure

Acoustic change complex responses were recorded across 32 channels using the actiCHamp Brain Products recording system (Brain Products GmbH, Inc., Munich, Germany) during passive listening to babble noise -/ba/with the same noise. The blind subjects sat in a comfortable chair reading a braille book. During recording, we encouraged the participants to stay still during the test with their heads and the elbows within a fixed range. We also instructed them not to move their wrist and fingers. The sighted subjects sat watching a muted, closed-captioned movie. A notch filter at 60 Hz was set to prevent powerline noise and the impedance of all scalp electrodes was kept <5 kΩ.

#### Data Processing

The collected data were analyzed using Brain vision analyzer version 2.0 (Brain Products GmbH, Inc., Munich, Germany). The band-pass filter was set at 0.1–60 Hz after removing eye blinks and body movement artifacts. In addition, independent component analysis was used to adjust for eye blinks. Babble noise –/ba/with same noise stimuli data were separated from 200 ms before stimulus presentation to 200 ms after stimulation based on the babble noise only stimulus presentation time. Baseline correction was performed using the interval before stimulation presentation, and potential averaging was performed. Using a semi-automatic peak detection algorithm in the Brain vision analyzer software, the largest negative deflection that occurred between 100 and 200 ms after stimulus onset was defined as the peak of N1. The peaks were visually inspected and manually adjusted if necessary.

### Statistical Analysis

To analyze the behavioral tests in this study, descriptive statistics, including the mean and standard deviation of each test, were determined for each group. Non-parametric tests were analyzed by confirming that each group did not follow a normal Kolmogorov–Smirnov distribution. The statistical comparisons between groups were made for each test with multiple independent *t-*tests or the Mann–Whitney test with Bonferroni’s correction. Within-group comparisons were made with multiple Wilcoxon signed-rank tests with Bonferroni’s correction. Correlation analyses were based on Pearson’s correlation coefficient.

## Results

### Behavioral Tests

#### Dichotic Digit Test

**FIGURE 1:**
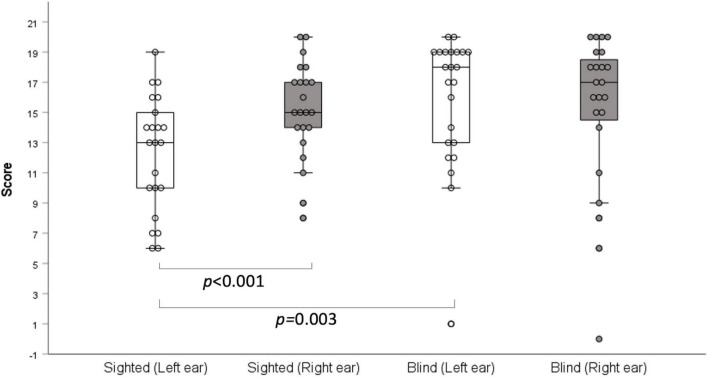
Dichotic three-digit test. Early-blind subjects had higher scores than the sighted subjects in the left ears (*p* = 0.003), but not in the right ears. A right-ear advantage was detected in the sighted subjects (*p* < 0.001), but not the early-blind subjects.

In the two-digit dichotic test, there was no significant difference between the two groups (right ear: *z* = –0.997, *p* = 0.319; left ear: *z* = –0.861, *p* = 0.389; Table 2). However, in the three-digit dichotic test, the early-blind subjects scored higher than the sighted subjects in the left ear (*z* = –2.979, *p* = 0.003, Bonferroni’s corrected α = 0.05/6 = 0.008; Table 2 and Figure 1) but not in the right ear. These results demonstrate that the right ear advantage was present in the sighted subjects (*z* = —3.715 *p* < 0.001, Bonferroni’s corrected α = 0.05/6 = 0.008), but not in the early-blind subjects (Figure 1).

**TABLE 2:** Statistical results of dichotic digit test and frequency pattern test.

Test		Blind	Sighted	*z*-score	*p*-value
Dichotic 2-digits	Right	19.04 ± 2.48	18.64 ± 2.22	–0.997	0.319
	Left	19.09 ± 1.41	18.50 ± 2.28	–0.861	0.389
Dichotic 3-digits	Right	15.22 ± 5.16	15.18 ± 3.22	–0.925	0.355
	Left	15.78 ± 4.51	12.27 ± 3.83	–2.979	0.003*
Frequency pattern					
Right	Humming	14.78 ± 0.52	13.50 ± 2.15	–2.722	0.006*
	Labeling	12.70 ± 2.27	11.14 ± 3.99	–1.172	0.241
Left	Humming	15.00 ± 0.00	14.09 ± 1.93	–2.899	0.004*
	Labeling	13.26 ± 2.24	10.36 ± 4.04	–2.755	0.006*

Values are presented as mean ± standard deviation. *p < 0.008 (Bonferroni’s corrected α = 0.05/6 = 0.008).

#### Frequency Pattern Test

**FIGURE 2:**
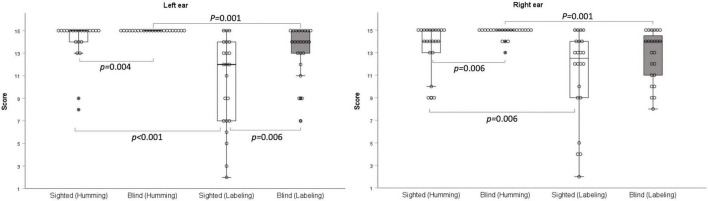
Frequency pattern test. With the humming response, early-blind subjects achieved significantly higher scores than sighted subjects (**right**: *p* = 0.006; **left** *p* = 0.004). With the labeling response, the scores were significantly higher in the early-blind subjects in the left ear only (*p* = 0.006).

The scores on the humming frequency pattern test were significantly greater for the early-blind subjects than the sighted subjects (left ear: *z* = –2.899, *p* = 0.004; right ear: *z* = –2.722, *p* = 0.006; Bonferroni’s corrected α = 0.05/6 = 0.008; Table 2 and Figure 2). All 23 early-blind subjects achieved full marks in the left frequency pattern test (15.0 ± 0.0). The results of the labeling test in the right ear did not differ significantly between the two groups, but the left ear score was significantly higher in the early-blind subjects (*z* = –2.755, *p* = 0.006, Bonferroni’s corrected α = 0.05/6 = 0.008; Table 2 and Figure 2). The results for the humming response were always higher than those for the labeling response, regardless of the side or group (*p* < 0.006, Bonferroni’s corrected α = 0.05/6 = 0.008; Figure 2).

#### Monosyllable Perception in Noise Test

**FIGURE 3:**
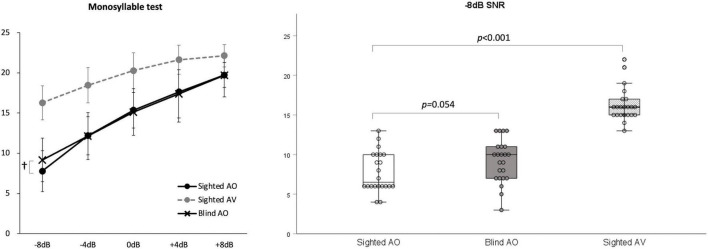
Monosyllable perception in noise test. At signal-to-noise ratio (SNR)s of –4, 0, +4, and +8 under auditory-only (AO) conditions, there were no significant differences between the early-blind and sighted subjects, but the early-blind subjects tended to do better than the sighted subjects at an SNR of –8 (*p* = 0.054). The scores of the sighted subjects under audio–visual (AV) conditions were much higher than those of the early-blind subjects and sighted subjects under the AO conditions (*p* < 0.001). †0.05 < *p* < 0.06.

The results at SNRs of –8, –4, 0, +4, and +8 under AO conditions did not differ significantly between the early-blind and sighted subjects, but tended to be better in the former group at an SNR of –8 dB (*z* = –1.9, *p* = 0.054; Table 3 and Figure 3). At all five SNRs, the scores of the sighted subjects under the AV condition were much higher than those of the early-blind subjects and the sighted subjects under the AO condition (*p* < 0.003, Bonferroni’s corrected α = 0.05/15 = 0.003; Table 3).

**TABLE 3:** Statistical results of the monosyllable perception in noise test.

SNR	AO		*z*-score	*p*-value^a^	AV	*z*-score	*p*-value^b^
				
	Blind	Sighted			Sighted		
–8 dB	9.17 ± 2.69	7.77 ± 2.54	–1.9	0.054^†^	16.27 ± 2.12	–5.731	<0.000*
–4 dB	12.13 ± 2.91	12.18 ± 2.38	–0.4	0.714	18.45 ± 2.20	–5.182	<0.000*
0 dB	15.13 ± 2.90	15.36 ± 2.22	–0.1	0.900	20.27 ± 2.23	–4.879	<0.000*
+4 dB	17.39 ± 3.00	17.64 ± 3.75	–0.4	0.698	21.64 ± 1.79	–4.787	<0.000*
+8 dB	19.70 ± 2.67	19.73 ± 1.55	–0.1	0.900	22.14 ± 1.39	–3.167	0.002*

*Values are presented as mean ± standard deviation.*

*SNR, signal-to-noise ratios; AO, auditory-only; AV, audio–visual.*

*^a^Comparison between blind and sighted subjects, ^†^Near-significant difference.*

^b^Multiple comparisons among the AO condition in blind subjects, the AO condition in sighted subjects, and the AV condition in sighted subjects, *p < 0.003 (Bonferroni’s corrected α = 0.05/15 = 0.003).

#### Digit Span Test

**FIGURE 4:**
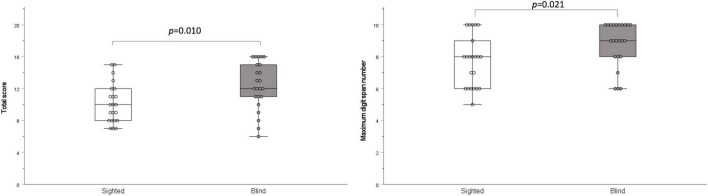
Digit span test. The total score and maximum digit number were significantly higher in the blind group than in the sighted group (*p* = 0.010 and *p* = 0.021, respectively).

The results of the digit span test were significantly higher in the early-blind subjects than in the sighted subjects (total score: *z* = –2.579, *p* = 0.010; maximum digit number: *z* = –2.306, *p* = 0.021; Figure 4).

#### Correlation Analyses

In the early-blind subjects, the total score on the digit span test correlated significantly with the score on the three-digit dichotic test in the right ear (*r* = 0.698, *p* < 0.001) and the left ear (*r* = 0.531, *p* = 0.011), and the maximum digit number on the digit span test also correlated significantly with the score on the three-digit dichotic test in the right ear (*r* = 0.735, *p* < 0.001) and the left ear (*r* = 0.612, *p* = 0.002). In the early-blind subjects, the total scores on the digit span test correlated significantly with the score on the humming frequency pattern test in the right ear (*r* = 0.451, *p* = 0.035), and the maximum digit number on the digit span test also correlated significantly with the score on the humming frequency pattern test in the right ear (*r* = 0.445, *p* = 0.038). However, there was no correlation between the result of the digit span test and the scores on the three-digit dichotic or frequency pattern tests in sighted subjects.

### Electrophysiological Results

**FIGURE 5:**
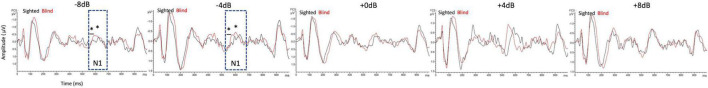
Comparisons of acoustic change complex (ACC) at the FC5 electrode between early-blind subjects and sighted subjects. The N1 peak amplitude originating from/ba/in babble noise was greater in early-blind subjects than in sighted subjects at SNRs -of -8 dB and –4 dB (*p* = 0.004 and *p* = 0.003, respectively, Bonferroni’s corrected α = 0.05/5 = 0.01). The latency of the N1 peak at the FC5 electrode was shorter in the early-blind subjects than in the sighted subjects under an SNR of –8 and –4 dB (*p* = 0.007 and *p* < 0.001 respectively, Bonferroni’s corrected α = 0.05/5 = 0.01). *Significant in the N1 peak amplitude; * Significant in the peak latency.

Figure 5 shows the grand mean ACC potentials in response to the signal change at the FC5 electrodes. The N1 peak amplitude originating from/ba/in babble noise was greater in the early-blind subjects than in the sighted subjects only at FC5 electrode under SNRs of –8 dB and –4 dB [*t*_(42)_ = –3.088, *p* = 0.004 and *t*_(42)_ = –3.112, *p* = 0.003 respectively, Bonferroni’s corrected α = 0.05/5 = 0.01]. The latency of the N1 peak at the FC5 electrode was shorter in the early-blind subjects than in the sighted subjects under an SNR of –8 and –4 dB [*t*_(42)_ = –2.827, *p* = 0.007 and *t*_(42)_ = –4.911, *p* < 0.001 respectively, Bonferroni’s corrected α = 0.05/5 = 0.01].

## Discussion

In the dichotic three-digit test, the early-blind subjects showed an equally high performance in both ears, indicating no ear advantage. By contrast, the sighted subjects displayed the typical right-ear advantage, consistent with the findings of another study (Jang et al., 2014), which assumes that the right ear connects directly to the left hemisphere, which is dominant in right-handed subjects. Therefore, the dichotic three-digit test results revealed better right hemispheric function or interhemispheric function in early-blind subjects than in sighted subjects. In other words, the symmetric performances of both ears (i.e., no right-ear advantage) in early-blind subjects are attributed to the enhanced function of the right hemisphere, which is dominantly connected with the left ear. This finding suggests evidence of plastic functional changes in the brain after long-term visual deprivation. Another explanation is that the education of early-blind subjects to read Braille with a two-handed technique (Hermelin and O’Connor, 1971) may affect symmetric performance in the dichotic test because the dichotic-listening ear advantage is known to be associated with the subject’s handedness (Hu and Lau, 2019). Although all early-blind subjects in the present study were right-handed, two-hand usage in early-blind subjects could functionally enhance the right hemisphere. In a previous study using a dichotic test with the pairwise presentation of CV syllables, the overall correct scores were higher in the early-blind group than in the sighted subjects, consistent with our data using a three-digit dichotic test (Hugdahl et al., 2004). However, those authors documented a right-ear advantage in early-blind subjects as in sighted subjects, implying that this difference was caused by the test materials (digits vs CV syllables).

In the frequency pattern test, the early-blind subjects performed better in the humming response than the sighted subjects. With the labeling response in words, the early-blinded subjects achieved higher scores than the sighted subjects when the stimuli were presented to the left ear. The left hemisphere plays a dominant role in speech and language, whereas the right hemisphere recognizes acoustic contours and patterns (Blumstein and Cooper, 1974). Therefore, the results for the humming response reflect the better function of the right hemisphere in early-blind subjects, and the results for the linguistic labeling response in the left ear imply greater transfer of the acoustic signals from the right hemisphere to the left hemisphere in early-blind subjects.

If the results of the two tests are combined and interpreted, blind subjects might have (1) better right hemisphere function; (2) greater transfer of auditory information from the right to the left hemisphere; or (3) better binaural integration than sighted subjects. Previous studies that evaluated the auditory performance of blind subjects examined various psychoacoustic abilities, such as pitch discrimination (Gougoux et al., 2004; Shim et al., 2019), temporal resolution (Stevens and Weaver, 2005; Shim et al., 2019), ultrafast speech comprehension (Hertrich et al., 2013), sound localization (Zwiers et al., 2001; Lewald, 2002; Gori et al., 2010, 2014; Finocchietti et al., 2015; Vercillo et al., 2016; Battal et al., 2020), and speech perception (Gougoux et al., 2009; Guerreiro et al., 2015; Shim et al., 2019), and those abilities are also affected by the function of the peripheral auditory system. Both the dichotic digit test and the frequency pattern test reflect the central auditory processing of a subject, with no influence from any mild to moderate damage in the peripheral auditory system (Musiek, 1994; Mukari et al., 2006). The results of the present study suggest that long-term visual deprivation reduces the natural advantage of the left hemisphere for speech perception by enhancing the function of right hemisphere and enhancing the transfer of information from the right hemisphere to the left hemisphere. An early neuroimaging study showed preferential activation of right occipital cortex during sound localization in early-blind subjects implying the auditory recruitment of right visual cortex (Weeks et al., 2000). This plastic change in favor of the right brain is consistent with the results of the present study.

Many previous studies have compared the speech perception abilities of early-blind subjects with those of sighted subjects, but the results varied according to the experimental setting. Several studies showed better speech perception in the early-blind group (Menard et al., 2009; Arnaud et al., 2018), whereas other studies found no behavioral differences between early-blind and sighted subjects (Gougoux et al., 2009; Guerreiro et al., 2015; Shim et al., 2019). In a previous study, we used two-syllable words and white noise and found no difference between the blind and sighted groups. However, in the present study, the test words were changed to monosyllabic words, which allow fewer redundancy cues in speech perception. Furthermore, babble noise presents more challenging interference to speech perception than white noise because it has a high time-evolving structure and is more similar to the target speech (Cooke, 2006). However, there were no differences between the early-blind subjects and sighted subjects under four high SNRs (–4 dB, 0 dB, +4 dB, and +8 dB). Only under the most severely degraded listening conditions (i.e., SNR of –8 dB) did the early-blind subjects tend to perform better than sighted subjects on monosyllabic perception in noise, although the difference was not significant. Therefore, it is still difficult to conclude whether there are differences in speech perception in noise between early-blind individuals and sighted individuals. The ACC responses to/ba/in babble noise recorded at the FC5 electrode showed significantly greater neuronal power at –8 and –4 dB SNRs in the early blind subjects than in the sighted subjects. Because the ACC stimuli were designed to detect speech perception, a significant difference might only be detected at the left-hemispheric electrode. These findings suggest that early-blind subjects could have an advantage in speech perception when the original speech cue is severely degraded, despite the inconclusive result on the behavioral test. The inconsistent results for speech perception in many previous studies, including ours, might be attributable to plastic changes that occur in the central auditory system after long-term visual deprivation, mainly involving the right hemisphere rather than the left hemisphere, which plays a dominant role in speech and language (Kimura, 1961; Blumstein and Cooper, 1974). The plasticity of the left hemisphere, which is indirectly affected by the interhemispheric transfer from the right hemisphere, might be limited. The better performance of sighted subjects under AV conditions than the performance of early-blind subjects or sighted subjects under AO conditions was entirely predictable. The multisensory integration of auditory and visual stimuli facilitates speech recognition in noise better than under unimodal conditions (Giard and Peronnet, 1999).

The digit span test is traditionally used worldwide to evaluate memory span, especially verbal working memory (Harris et al., 2013). Our results for the digit span test show that the early-blind subjects had a significantly larger memory span than the sighted subjects. Previous research findings for working memory in early-blind subjects compared with those of sighted subjects have been inconclusive. Several studies found no differences between blind and sighted subjects (Cornoldi and Vecchi, 2000; Swanson and Luxenberg, 2009), whereas other studies have reported that blind subjects have an advantage over their sighted peers in working memory tasks, including the digit span test (Rokem and Ahissar, 2009; Withagen et al., 2012), and the word memory test (Raz et al., 2007). Raz et al. (2007) argued that early-blind subjects have trained themselves in serial strategies to compensate for the lack of visual information, and that their perception of space is likely to be highly dependent on memory. This superior ability could be attributed to actual brain reorganization in blind subjects, whose brains become more adapted to spatial, sequential, and verbal information (Cornoldi and Vecchi, 2000). In the present study, the results of the digit span test correlated with the score on the three-digit dichotic test and the score on part of the frequency pattern test in the early-blind subjects, but not in the sighted subjects. These results imply that the advantages of early-blind subjects in the dichotic digit test and frequency pattern test may be partly dependent on their superior working memory.

In conclusion, early-blind subjects are advantaged in dichotic listening and temporal sequencing ability. They also tend to have an advantage in monosyllable perception in very noisy backgrounds. These advantages may be attributable to the enhanced activity of the central auditory nervous system, especially the right hemisphere function, and the transfer of auditory information between hemispheres.

## Data Availability Statement

The original contributions presented in the study are included in the article/Supplementary Material, further inquiries can be directed to the corresponding author.

## Ethics Statement

The studies involving human participants were reviewed and approved by Institutional Review Board of Nowon Eulji Medical Center. The patients/participants provided their written informed consent to participate in this study.

## Author Contributions

HS: conceptualization and funding acquisition. HS and EB: experiment design. EB: experiment performance and data preparation and analysis. HJ and EB: methodology. HJ and HS: critical review. All authors contributed to the writing manuscript and approved the submitted version.

## Conflict of Interest

The authors declare that the research was conducted in the absence of any commercial or financial relationships that could be construed as a potential conflict of interest.

## Publisher’s Note

All claims expressed in this article are solely those of the authors and do not necessarily represent those of their affiliated organizations, or those of the publisher, the editors and the reviewers. Any product that may be evaluated in this article, or claim that may be made by its manufacturer, is not guaranteed or endorsed by the publisher.
