# Supplementary material for: Enhanced Dichotic Listening and Temporal Sequencing Ability in Early-Blind Individuals
Source: Front Psychol. 2022 May 10;13:840541. doi: 10.3389/fpsyg.2022.840541 (PMC9127502; doi:10.3389/fpsyg.2022.840541)
Supplement: Supplementary file 1 [file Table_1.DOCX]

Supplementary Material 1

**Digit Span Test**

|  | |  | | | | | | | | | | **Response** | |
| --- | --- | --- | --- | --- | --- | --- | --- | --- | --- | --- | --- | --- | --- |
|  |  |  |  |  |  |  |  |  |  |  |  | **Correct** | **Incorrect** |
|  | 3-2 | **8** | **1** | **3** |  |  |  |  |  |  |  | + | - |
|  | 4-1 | **7** | **4** | **8** | **2** |  |  |  |  |  |  | + | - |
|  | 4-2 | **4** | **8** | **3** | **7** |  |  |  |  |  |  | + | - |
|  | 5-1 | **3** | **8** | **6** | **7** | **9** |  |  |  |  |  | + | - |
|  | 5-2 | **7** | **5** | **9** | **4** | **6** |  |  |  |  |  | + | - |
|  | 6-1 | **6** | **1** | **5** | **4** | **2** | **7** |  |  |  |  | + | - |
|  | 6-2 | **3** | **9** | **2** | **8** | **1** | **6** |  |  |  |  | + | - |
|  | 7-1 | **2** | **10** | **4** | **8** | **3** | **6** | **1** |  |  |  | + | - |
|  | 7-2 | **5** | **9** | **1** | **2** | **6** | **4** | **7** |  |  |  | + | - |
|  | 8-1 | **3** | **5** | **1** | **6** | **4** | **10** | **2** | **5** |  |  | + | - |
|  | 8-2 | **6** | **1** | **9** | **3** | **5** | **2** | **7** | **4** |  |  | + | - |
|  | 9-1 | **3** | **9** | **8** | **1** | **10** | **4** | **6** | **5** | **2** |  | + | - |
|  | 9-2 | **9** | **5** | **8** | **2** | **4** | **3** | **1** | **7** | **6** |  | + | - |
|  | 10-1 | **2** | **5** | **1** | **10** | **6** | **9** | **4** | **7** | **3** | **8** | + | - |
|  | 10-2 | **4** | **2** | **9** | **6** | **10** | **3** | **7** | **1** | **8** | **5** | + | - |
|  | | | | | | | | | | | | **Total** | **/ 16** |
